# Association of self-rated health with chronic disease, mental health symptom and social relationship in older people

**DOI:** 10.1038/s41598-021-94318-x

**Published:** 2021-07-19

**Authors:** Hongling Yang, Qin Deng, Qingshan Geng, Yanfei Tang, Jun Ma, Weitao Ye, Qiangsheng Gan, Rehemutula Rehemayi, Xiaoli Gao, Chunyan Zhu

**Affiliations:** 1 Guangzhou Women and Children’s Medical Centre, Guangzhou Medical University, Guangzhou, China; 2 Pharmacy Department, Dongguan Qishi Hospital, Dongguan, China; 3 Guangdong General Hospital, Guangdong Academy of Medical Science, Guangzhou, China; 4 Zhongshan Dachong Community Health Service Centre, Zhongshan, China; 5 Department of Prevention Medicine, School of Public Health, Guangzhou Medical University, Guangzhou, China; 6 Faculty of Dentistry & Saw Swee Hock School of Public Health, National University of Singapore, Singapore, Singapore

**Keywords:** Health care, Risk factors

## Abstract

Chronic disease, mental health symptoms and poor social relations are reported common causes for poor self-rated health in older people. To assess the co-occurrence rate of chronic diseases, poor mental health and poor social relationships in older people, and determine their association with self-rated health. 6,551 older people in Zhongshan, China, participated a large health surveillance program were randomly selected and questioned about their SRH, chronic conditions, mental health symptoms and social relationships. The association between self-rated health and chronic conditions, poor mental health, social relationships, and their co-occurrence were analyzed. 56.4% of participants reported poor self-rated health. 39.1% experienced at least one chronic disease. 29.0% experienced one or more mental health symptoms; 19.5% experienced at least one poor social relationship. 7.8% had co-occurrence of chronic diseases, mental health problems, and poor social relationships. Logistic regressions showed that poor self-rated health was associated with chronic diseases, poor mental health, poor social relationships and their co-occurrence. The findings indicate the importance of managing chronic disease, poor mental health and poor social relationships for older people.

## Introduction

Self-rated health is a commonly used measure for subjectively perceived health^1^. It provides an overview of an individual’s health status and it is associated with future health outcomes, including mortality and subclinical aspects of personal health^1,2^.

Self-rated health is associated with various health predictors and outcomes, such as sociodemographic characteristics, lifestyle factors, physical health status, mental health status, and functional limitations^3^. Self-rated health decreases with physical well-being, and significant differences are observed between those with the best and worst levels of social well-being^4^. A study carried out among older people in Shanghai showed that the main determinants of self-rated health among older people included living conditions, health risk behavior, social support, health status, and the economic status of the neighborhood^5^.

Previous research has largely focused on the effect of individual predictors of self-rated health, despite the high prevalence of their co-occurrence among older people. The ageing process results in an increase in the comorbidity of chronic diseases, disability, reduced levels of functioning, and mental health problems. It was reported that more than 50% of adults aged 55 years and older had at least two chronic conditions^3^, and somatic complaints are highly associated with mental health symptoms^6^.

In China, the number of elderly people is increasing^7^. Progressive ageing has a profound impact on the physical, social and mental health of the population. Chronic diseases, poor mental health and poor social relationships affect older people^8^. Among the elderly over the age of 60, 23.8% had poor mental health status and 68.7% suffered from two or more chronic diseases^8^. The high prevalence of chronic diseases, poor mental health and poor social relations among older people in China raises questions as to whether, and to what extent, these conditions account for their poor self-rated health.

Concrete evidence indicates that the risk of poor self-rated health increases with the number of chronic diseases^9^, mental health symptoms^10^, and social support^11^. However, there is a lack of research concerning the association between self-rated health and the co-occurrence of chronic diseases, mental health symptoms, and poor social relationships, especially among older people. Furthermore, little attention has been paid to the correlation between chronic diseases, mental health symptoms, and poor social relationships in terms of self-rated health among community-dwelling older people in China.

Zhongshan is a city that is located at the southern tip of the Chinese mainland and the eastern bank of the mouth of the Pearl River. In less than 30 years, Zhongshan has grown from a traditional agricultural town into a modern metropolis^12^. Older people in Zhongshan can be regarded as a typical population that has aged under the reform and opening-up policy in China, which started in the late 1970s. Urbanization and subsequent changes in lifestyles, family patterns, and social relationships are likely to have profound impacts on their health.

The current study, centered on a population-based sample of older adults in Zhongshan, South China, and it aimed to (i) determine the rate of chronic diseases, mental health symptoms and poor social relationships, as well as their co-occurrence, and (ii) to investigate the association between these factors and self-rated health.

## Results

### Sample characteristics

In total, 6,551 older people participated in this study. The mean age was 70.2 (S.D. = 8.1) years. The majority of the participants (82.0%) was below primary school or had only completed primary education, while 9.4% lived alone, and 37.6% and 57.3% reported good economic conditions and housing conditions, respectively (Table 1).

**Table 1:** Socio-demographic characteristics of respondents (n = 6 551).

	n	%
Age (year)		
Mean ± SD	70.2 ± 8.1	
Min–max	60–104	
Age group (year)		
60–69	3549	54.2
70–79	2067	31.5
≥ 80	935	14.3
Sex		
Male	3539	54.0
Female	3012	46.0
Marital status*		
Married	4727	72.2
Unmarried	125	1.9
Separated /widowed/divorced	1694	25.9
Education level*		
Below primary school	1904	29.1
Primary	3464	53.0
Secondary and higher	1172	17.9
Living arrangement		
With other person(s)	5935	90.6
Alone	616	9.4
Economic conditions		
Good	2467	37.6
Fair	3437	52.5
Poor	647	9.9
Housing conditions		
Good	3757	57.3
Fair	2540	38.8
Poor	254	3.9

*Data of some participants were missing.

Among the 6,551 participants, 56.4% reported poor self-rated health; 28.6% suffered from one chronic disease and 10.5% reported two or more chronic diseases; 29.0% experienced one mental health symptom and 44.3% reported two or more symptoms; 19.5% experienced at least one type of poor social relationship; 34.8% experienced two co-occurrent conditions (e.g., chronic disease and/or mental health symptom and/or poor social relationships) and 7.8% reported three co-occurrent conditions (chronic disease, mental health symptoms, and poor social relationships). Approximately half of participants (47.7%) reported fair or poor sleep; 18.4% required medication to support their daily life; and 66.8% experienced at least one limitation in their activities (Table 2).

**Table 2:** Prevalence of reported health conditions (n = 6 551).

	n	%
Self-rated health		
Good	2853	43.6
Poor	3698	56.4
Chronic disease		
At least one chronic condition	2558	39.1
1 Chronic condition	1873	28.6
≥ 2 Chronic conditions	685	10.5
Mental health symptom*		
1 symptom	1897	29.0
≥ 2 symptoms	2903	44.3
Social relation*		
≥ 1 poor relationships	1278	19.5
Chronic disease, mental health symptom and poor social relation combined*		
One present	2626	41.1
Two present	2224	34.8
All three present	496	7.8
Appetite*		
Good	3978	60.6
Fair	2446	37.3
Poor	140	2.1
Sleep quality*		
Good	3428	52.3
Fair	2650	40.5
Poor	472	7.2
Dependent on medication*		
Not at all or seldom	4476	68.3
Sometimes	868	13.3
Often or always	1205	18.4
Activity limitation*		
Doing housework	3775	57.6
Stair-climbing	3592	54.8
Stooping, kneeling or crouching	3288	50.2
Walking	3536	54.0
1 limitation	613	9.4
≥ 2 limitations	3758	57.4

*Data of some participants were missing.

**Figure 1:**
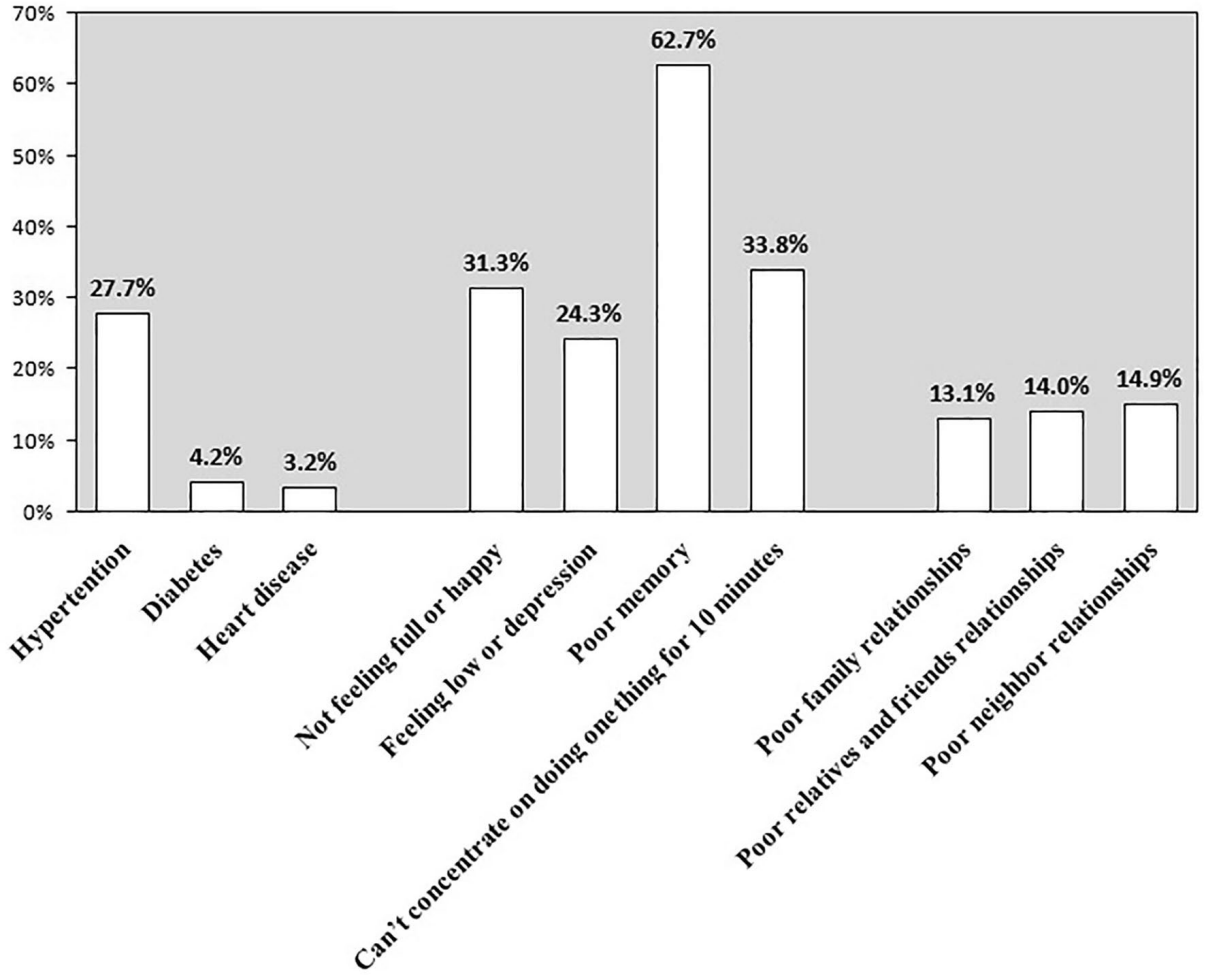
Proportion of chronic diseases, mental health symptoms and poor social relations among the participants.

Figure 1 shows the number and proportion of participants with various physical diseases, psychological states, and social relationships. Hypertension (27.7%), poor memory (62.7%) and poor neighbor relationships (14.9%) were the most commonly reported problems.

### Association between self-rated health and chronic disease, mental health symptoms, and poor social relationships

The bivariate analysis showed that, compared with participants who reported good self-rated health, those who reported poor self-rated health had a higher prevalence of chronic disease (52.8% vs. 21.2%, *P* < 0.001), mental health symptoms (87.9% vs. 58.5%, *P* < 0.001), poor social relationships (26.8% vs. 10.2%, *P* < 0.001), and the co-occurrence of chronic disease, mental health symptoms, and poor social relationships (12.4% vs. 1.7%, *P* < 0.001). Poor self-rated health was significantly associated with the three leading chronic conditions (i.e., hypertension, diabetes and heart disease), mental health symptoms (feeling unsatisfied with life, feeling low or depressed, poor memory and being unable to concentrate for 10 min) and poor social relationships (with family, friends, and neighbors) (Ps < 0.05) (Table 3).

**Table 3:** Bivariate analysis for relationship of chronic disease, mental health symptom and poor social relation with self-rated health.

	Self-rated health		OR (95%CI)
	Good (%)	Poor (%)	
No of chronic diseases			
0	2248 (78.8)	1745 (47.2)	1.00
1	506 (17.7)	1367 (37.0)	3.48 (3.09–3.92)**
≥ 2	99 (3.5)	586 (15.8)	7.63 (6.11–9.52)**
No of mental health symptoms			
0	1152 (41.5)	436 (12.1)	1.00
1	914 (32.9)	983 (27.2)	2.84 (2.47–3.28)**
≥ 2	712 (25.6)	2191 (60.7)	8.13 (7.08–9.34)**
No of poor social relations			
0	2557 (89.8)	2705 (73.2)	1.00
≥ 1	290 (10.2)	988 (26.8)	3.22 (2.80–3.71)**
No of chronic disease, mental health symptomand poor social relation combined			
None present	876 (31.6)	170 (4.7)	1.00
One present	1353 (48.8)	1265 (35.1)	4.82 (4.02–5.78)**
Two present	497 (17.9)	1725 (47.8)	17.89 (14.76–21.68)**
Three present	47 (1.7)	448 (12.4)	49.12 (34.87–69.18)**
Chronic disease^#^			
Hypertension	428 (15.0)	1387 (37.5)	3.40 (3.01–3.84)**
Diabetes mellitus	48 (1.7)	228 (6.2)	3.84 (2.80–5.26)**
Cardiac disease	29 (1.0)	182 (4.9)	5.04 (3.40–7.48)**
Mental health symptom^#^			
Not feeling satisfied with life	448 (15.7)	1604 (43.4)	4.11 (3.65–4.64)**
Feeling downhearted or depressed	396 (13.9)	1196 (32.4)	2.97 (2.61–3.37)**
Poor memory	1271 (45.6)	2834 (78.3)	4.30 (3.85–4.79)**
Can’t concentrate on doing one thing for 10 min	559 (19.6)	1653 (44.8)	3.33 (2.97–3.72)**
Social relation^#^			
Poor family relationships	155 (5.4)	706 (19.1)	4.11 (3.43–4.92)**
Poor friends relationships	171 (6.0)	746 (20.2)	3.97 (3.33–4.72)**
Poor neighbor relationships	194 (6.8)	784 (21.2)	3.69 (3.13–4.35)**

^#^Both counts and percentages are based upon weighted inputted values.

**The reference category is subjects without the specified condition, *P* < 0.01.

The logistic regression models, which included self-rated health (good vs. poor) as the dependent variable, demonstrated that poor self-rated health, after adjusting for other background characteristics, was significantly associated with chronic disease, mental health symptoms, and poor social relations (Ps < 0.05). The probability of poor self-rated health was higher among individuals with more than one chronic disease, mental health symptoms, and poor social relationships (odds ratio (OR): 4.27, 95% confidence interval (CI): 3.24–5.62; 2.75, 2.31–3.29; and 1.35, 1.13–1.62, respectively). In addition, poor economic conditions (OR 1.53; 95% CI: 1.30–1.79), poor housing conditions (1.78; 1.15–2.77), poor appetite (3.84; 1.82–8.12), poor sleep (1.95; 1.46–2.61), medication dependence (3.51; 2.82–4.37), and activity limitations (2.17; 1.87–2.52) were significantly associated with poor self-rated health, whereas participants with “a secondary or higher” education level (0.79; 0.64–0.97) and those who “lived alone” (0.73; 0.57–0.93) were significantly associated with good self-rated health (Table 4).

**Table 4:** Multivariate analysis of the relationship of chronic disease, mental health symptom and poor social relation with self-rated health.

	Model 1	Model 2	Model 3	Model 4
	OR(95% CI)	OR(95% CI)	OR(95% CI)	OR(95% CI)
No of chronic diseases				
0	1.00	1.00	1.00	1.00
1	3.73 (3.27–4.26)**	4.05 (3.53–4.65)**	4.18 (3.64–4.80)**	2.67 (2.27–3.15)**
≥ 2	7.23 (5.72–9.15)**	7.92 (6.22–10.09)**	8.31 (6.52–10.59)**	4.27 (3.24–5.62)**
No of mental health symptoms				
0	1.00	1.00	1.00	1.00
1	2.60 (2.24, 3.03)**	2.33 (1.99–2.72)**	2.32 (1.98–2.71)**	1.80 (1.52–2.13)**
≥ 2	6.79 (5.85, 7.88)**	5.42 (4.63–6.34)**	5.20 (4.43–6.09)**	2.75 (2.31–3.29)**
No of poor social relations				
0	1.00	1.00	1.00	1.00
≥ 1	2.36 (2.01, 2.77)**	1.95 (1.65–2.30)**	1.84 (1.55–2.18)**	1.35 (1.13–1.62)**
Age group (year)				
60–69		1.00	1.00	1.00
70–79		1.09 (0.95–1.25)	1.11 (0.96–1.27)	0.89 (0.75–1.03)
≥ 80		1.15 (0.94–1.39)	1.16 (0.95–1.42)	0.81 (0.65–1.01)
Sex				
Male		1.00	1.00	1.00
Female		1.15 (1.02–1.31)*	1.16 (1.02–1.31)*	1.03 (0.89–1.18)
Marital status				
Married		1.00	1.00	1.00
Unmarried		0.92 (0.59–1.44)	1.04 (0.65–1.67)	0.92 (0.55–1.52)
Widowed/divorced		0.91 (0.78–1.05)	0.96 (0.82–1.13)	0.93 (0.79–1.11)
Education level				
Below primary school		1.00	1.00	1.00
Primary		0.98 (0.85–1.13)	0.98 (0.84–1.13)	0.95 (0.81–1.12)
Secondary and higher		0.87 (0.72–1.06)	0.88 (0.73–1.07)	0.79 (0.64–0.97)*
Economic conditions				
Good		1.00	1.00	1.00
Fair		2.48 (2.18–2.81)**	1.85 (1.60–2.15)**	1.53 (1.30–1.79)**
Poor		3.07 (2.43–3.89)**	2.06 (1.57–2.69)**	1.74 (1.30–2.33)**
Housing conditions				
Good			1.00	1.00
Fair			1.82 (1.57–2.11)**	1.65 (1.41–1.94)**
Poor			1.96 (1.31–2.94)**	1.78 (1.15–2.77)**
Living arrangement				
With other person(s)			1.00	1.00
Alone			0.74 (0.59–0.93)*	0.73 (0.57–0.93)*
Appetite				
Good				1.00
Fair				2.15 (1.84–2.51)**
Poor				3.84 (1.82–8.12)**
Sleep quality				
Good				1.00
Fair				2.36 (2.04–2.74)**
Poor				1.95 (1.46–2.61)**
Dependent on medication				
Not at all or seldom				1.00
Sometimes				2.60 (2.10–3.22)**
Often or always				3.51 (2.82–4.37)**
No of activity limitations				
0				1.00
1				1.48 (1.18–1.86)**
≥ 2				2.17 (1.87–2.52)**

*The reference category is subjects without the specified condition, *P* < 0.05.

**The reference category is subjects without the specified condition, *P* < 0.01.

The results of the logistic regression models, which included self-rated health (good vs. poor) as the dependent variable also demonstrated that, compared with participants who reported good self-rated health, those who reported a co-occurrence of chronic disease, mental health symptoms, and poor social relationships were significantly more likely to report poor self-rated health. In addition, from the first factor onward, a cumulative and negative correlation was found between co-occurring multimorbidity and with self-rated health (OR of one present: 2.35, 95% CI: 1.92–2.87; two present: 5.04, 4.04–6.28; three present: 8.47, 5.76–12.45). The association of the background characteristics with self-rated health was the same as that reported in Table 4 (Table 5).

**Table 5:** Multivariate analysis of the relationship of co-occurrence of chronic disease and mental health symptom and poor social relation with self-rated health.

	Model 1	Model 2	Model 3	Model 4
	OR(95% CI)	OR(95% CI)	OR(95% CI)	OR(95% CI)
No of chronic disease and mental symptom and poor social relation combined				
None present	1.00	1.00	1.00	1.00
One present	4.82 (4.02–5.78)**	4.11 (3.42–4.95)**	4.04 (3.36–4.87)**	2.35 (1.92–2.87)**
Two present	17.89 (14.76–21.68)**	14.48 (11.88–17.64)**	14.25 (11.68–17.39)**	5.04 (4.04–6.28)**
Three present	49.12 (34.87–69.18)**	33.15 (23.36–47.03)**	31.80 (22.36–45.22)**	8.47 (5.76–12.45)**
Age group (year)				
60–69		1.00	1.00	1.00
70–79		1.16 (1.01–1.33)*	1.18 (1.03–1.35)*	0.90 (0.77–1.05)
≥ 80		1.33 (1.10–1.60)**	1.34 (1.10–1.62)**	0.86 (0.69–1.07)
Sex				
Male		1.00	1.00	1.00
Female		1.16 (1.03–1.32)*	1.16 (1.03–1.32)*	1.03 (0.89–1.18)
Marital status				
Married		1.00	1.00	1.00
Unmarried		0.90 (0.58–1.40)	1.03 (0.65–1.64)	0.92 (0.55–1.52)
Widowed/divorced		0.94 (0.81–1.09)	1.00 (0.86–1.17)	0.96 (0.81–1.14)
Education level				
Below primary school		1.00	1.00	1.00
Primary		0.98 (0.85–1.13)	0.98 (0.84–1.13)	0.95 (0.81–1.11)
Secondary and higher		0.88 (0.73–1.06)	0.90 (0.74–1.09)	0.79 (0.64–0.98)*
Economic conditions				
Good		1.00	1.00	1.00
Fair		2.39 (2.12–2.70)**	1.80 (1.56–2.07)**	1.51 (1.29–1.76)**
Poor		3.27 (2.61–4.10)**	2.20 (1.69–2.86)**	1.79 (1.34–2.38)**
Housing conditions				
Good			1.00	1.00
Fair			1.76 (1.53–2.03)**	1.61 (1.37–1.88)**
Poor			1.88 (1.26–2.79)**	1.71 (1.11–2.64)*
Living arrangement				
With other person(s)			1.00	1.00
Alone			0.72 (0.58–0.90)**	0.70 (0.55–0.90)**
Appetite				
Good				1.00
Fair				2.12 (1.82–2.47)**
Poor				3.50 (1.67–7.34)**
Sleep quality				
Good				1.00
Fair				2.34 (2.02–2.71)**
Poor				2.08 (1.56–2.77)**
Dependent on medication				
Not at all or seldom				1.00
Sometimes				2.94 (2.39–3.62)**
Often or always				4.45 (3.64–5.45)**
No of activity limitations				
0				1.00
1				1.42 (1.14–1.77)**
≥ 2				2.26 (1.96–2.62)**

*The reference category is subjects without the specified condition, *P* < 0.05.

**The reference category is subjects without the specified condition, *P* < 0.01.

## Discussion

Using a large representative sample with a high response rate, the present study demonstrated that a significant association between poor self-rated health and participants who experienced chronic diseases, mental health symptoms, and poor social relationships. The probability of reporting poor self-rated health was higher among participants who experienced more than one chronic disease, mental health symptoms, and poor social relationships. Participants who experienced the co-occurrence of chronic diseases, mental health symptom, and poor social relationships were significantly more likely to report poor self-rated health, and from the first factor onward, co-occurring multimorbidity was cumulatively and negatively correlated with self-rated health among the older population.

In this study population, the prevalence of chronic diseases, mental health symptoms, and poor social relationships was 39.1%, 73.3%, and 19.5% respectively. Moreover, the co-occurrence of two and three problems (i.e., chronic diseases, mental health symptoms and poor social relationships) was 34.8% and 7.8%, respectively. A study carried out in Denmark showed that 47.6% of older people aged 60–74 years old reported no chronic diseases, and clinical assessments revealed that 30.2% of the participants suffered from none of the chronic diseases investigated^13^. St John et al. found that among adults aged 65 years or older in Canada, 13.3% of those living in urban areas and 8.9% of those living in rural regions developed depressive symptoms^10^. The current study showed a relatively low rate of reported chronic diseases and a high rate of mental health symptoms. The low prevalence of self-reported chronic diseases may be due to the low education level of the participants, a limited knowledge of diseases, and a reluctance to report illness. The high rate of mental health symptoms was likely due to the data collection methodology used in this study, such as the inclusion of memory and concentration, as well as the general mental health symptom, such as depression or anxiety symptoms.

In the present study, the prevalence of poor self-rated health was 56.4%. Reports of poor self-rated health among older people varies among different studies. For example, a study conducted in Spain found that 39.7% of older people aged 65 years old and over reported positive self-rated health^14^. In another study of 185 community-dwelling Icelanders aged 65–88 years old, 45% rated their health as good, 37% reported fair or poor health, and 18% stated that their health was very good or excellent^3^. In China, a study revealed that 68.0% of middle-aged and older people (aged 50 to 70 years) in Beijing and Shanghai reported poor or fair self-rated health^15^.

According to the World Health Organization (WHO) definition, health is a state of complete physical, mental and social well-being, and it does not merely describe the absence of disease or infirmity^16^. Correspondingly, we included all of the three critical dimensions of health in our analyses, namely, self-rated chronic diseases, mental health symptoms and social relationships. The absence of chronic diseases is a key measure of successful aging^17^. Our results showed that, chronic diseases, and the presence of one or more chronic diseases, in particular, were a significant predictor of poor self-rated health. This result is similar to the findings of previous studies, both in China and beyond^5,18^. Our results also revealed that self-rated health was strongly associated with mental health symptoms and poor social relationships, after adjusting for sociodemographic variables, living arrangements, sleep quality, medication, and activity limitations. A recent study which investigated community-dwelling Icelanders aged 65–88 years old also found a robust relationship between poor self-rated health and depressive symptoms adjusting for gender^3^. Another study found an association between low social network involvement and poor self-rated health among older men, whereas perceptions of low social support were associated with poor self-rated health among older women^19^. An association was also found between poor self-rated health and social capital, such as a lack of personal support and the absence of experiences that caused the participants to feel a sense of pride, even after controlling for prominent health-related factors including disability pensions, ethnicity, and financial stress^20^.

In this study, the most significant finding was the association between self-rated health and the co-occurrence of chronic disease, mental health symptoms, and poor social relationships, as this association was the strongest and it was independent. It is notable that older people who experienced multiple chronic diseases, mental health symptoms and poor social relationships reported poor self-rated health. The social relationships may not directly describe health status, but they influence the likelihood of different objective health conditions being used as a basis for self-ratings. In line with the views of WHO, the findings of the current study highlighted the multidimensionality of health. Our results are also consistent with previous studies. Sheridan et al. found that co-existing chronic conditions and high depressive symptoms were associated with a higher probability of reported prospective disability in relation to daily living activities and poor self-rated health^21^. The results of Quiñones et al. showed a combination of depression and/or cognitive impairment with multimorbidity was associated with a substantially higher risk of prospective disability than that observed in the case of a combination of somatic conditions only^22^. A study carried out among older Chinese Americans showed that family cohesion and support moderated the relationship between acculturation and self-rated health, and family members and family support were associated with well-being^23^. By employing a large sample population-based study and controlling for a wide range of background characteristics, this study contextualized and elucidated the above conclusions. Most importantly, our findings highlighted the potential multiplicative effect of co-occurring multimorbidity, mental health symptoms, and poor social relationships on poor self-rated health.

The role of age and sex in self-health evaluations remains controversial. In contrast with the findings of some previous studies, we found no evidence of a significant correlation between age, sex, and self-rated health^5,14^. However, our results are in line with another study, which found that the self-rated health of older people aged over 56 years old was not affected by age or gender^24^. Moreover, we found that participants who lived alone reported good self-rated health, while participants who experienced activity-related limitations reported poor self-rated health. This result seems reasonable, because older people who have no mobility limitations and who perceive their health as good tend to live alone (independently). It can be expected that older people who live alone in the community represent a sample of healthier people. The results also highlighted that people who live autonomously and independently rated their health more positively, regardless of access to family support and social agencies^25^. We found a significant correlation between poor appetite and self-rated health. Poor appetite is a common problem among older people. It can contribute to weight loss and, nutritional deficiencies, and is associated with poor healthcare outcomes, including increased morbidity and mortality^26^. Although a reduction in energy intake is largely due to physiological changes that occur as a result of the ageing process, it may predispose older people to psychological symptoms (e.g., depression), social issues (e.g., poverty, loneliness and social isolation), and physical problems (e.g., common medical conditions and medication dependence)^27^. The findings of the current also confirmed some well-established risk factors of self-rated health, including poor economic conditions, poor housing conditions, poor sleep, and a low education level.

Collectively, the results of this study provide several novel insights into the association between self-rated health and the co-occurrence of chronic diseases, mental health symptoms and poor social relationship. Our results highlighted the importance of detecting and managing chronic diseases, mental health symptoms and social relationships among older people, as well as the co-occurrence for improving the health outcomes of this population.

Several limitations in this study warrant further comment. The data related to mental health, cognition, and activity-related limitations were obtained using the self-reporting method, rather than referring to the diagnoses of clinicians or clinical diagnostic scales. The self-reported nature of all of the data may have introduced response bias. However, the results of the internal consistency analysis of the questionnaire showed that the questionnaire had good reliability, and Cronbach’s alpha for mental health symptoms, poor social relationships, and activity-related limitation were 0.826, 0.884 and 0.915, respectively. Although some older people declined to take part in this study, the response rate was high. Therefore, we do not believe that the results were affected by non-respondents.

## Conclusion

Our research further identified that the co-occurrence of chronic diseases, mental health symptoms, and poor social relationships was associated with poor self-rated health, while co-occurring multimorbidity, mental health symptoms, and poor social relationships were cumulatively and negatively correlated with self-rated health. It is essential to develop specific interventions targeting lifestyle patterns, mental health and social relationships to prevent chronic morbidity and improve older people’s perceived health.

## Methods

### Sampling and recruitment

In this study, the sample included participants from the community health surveillance which was conducted in Zhongshan city in Guangdong, China, in 2011. The surveillance, which was carried out every 5 years, was cross-sectional with representative samples, and it therefore facilitated a better understanding of the major health problems and their related factors.

The participants were selected on the basis of the following criteria: (1) 60 years or older; (2) held permanent household registration in Zhongshan city; (3) were living in Zhongshan city when the study was carried out; and (4) were able to answer questions or complete the questionnaire independently.

A multistage sampling method was employed to select a representative sample of residents. The sampling frame was based on the household registration system. First, 30% of the districts/towns were randomly sampled from all 24 districts/towns in Zhongshan city. Second, 30% of the streets/villages were randomly sampled from each selected district/town. Then, 50% of the families were randomly sampled from each selected street/village. To guarantee an adequate sample size, a replacement method was adopted. In the event that a sampled family could not be contacted after three attempts, the nearest family was selected as substitute. A total of 19,086 households were recruited, with a substitution rate of 9.4%. The final sample included 43,028 participants, of which 7,029 were aged 60 years old or over. Among these 7,029 older participants, 6,551 completed the survey, including 3,539 (54.0%) males and 3,012 (46.0%) females. The response rate was 93.2%.

The study was approved by the research ethics review committee of Guangzhou Medical University. Written informed consents were obtained from all participants. All research processes were performed in accordance with the requirements of the ethics committee.

### Data collection

The researchers carried out home visits to approach the participants who were then requested to complete a one-on-one face-to-face interview using structured questionnaires. Data related to the participants’ demographics, socioeconomic status, self-rated health, chronic diseases, psychological states, social relationships, medication, and activity limitations were collected. The internal consistency analysis of the questionnaire showed that the questionnaire had good reliability, and Cronbach’s alpha for mental health symptoms, poor social relationships, and activity-related limitation were 0.826, 0.884 and 0.915, respectively. The interviewers included undergraduate or postgraduate (master’s) students at Guangzhou Medical University. Standard procedures were established to guide the recruitment, training, and supervision of the interviewers.

Self-rated health was measured by posing the following question: “During the past 4 weeks, how would you rate your health? Would you say, very good, good, fair, bad or very bad?”. Among 6551 participants completed the surveys, 441 (6.7%), 2412 (36.8%), 3060 (46.7%), 598 (9.1%) and 40 (0.6%) reported very good, good, fair, bad and very bad, respectively. The responses were collapsed into two categories, i.e., good (very good, good) and poor (fair, bad, very bad)^28^.

Information concerning chronic conditions was obtained using a checklist of chronic illnesses extracted from the International Diseases Classification (ICD-10), which included 300 common diseases. Participants were asked whether they have ever been diagnosed by a registered medical doctor as having of the stated physical health problems. If the participant’s illness did not on the list, the name of the specific illness was recorded.

The mental health status of the participants was measured by posing the following 4 questions: “Did you consider your life as happy and fulfilling in the past four weeks?” (Possible answers: 1 all the time/2 most of the time/3 sometimes/4 occasionally/5 never); “Did you feel downhearted or depressed in the past four weeks?” (Possible answers: 1 never/2 occasionally/3 sometimes/4 most of the time/5 all the time); “How has your memory been during the past four weeks” (Possible answers: 1 very good/2 good/3 fair/4 bad/5 very bad); and “Were you able to concentrate on whatever you were doing for 10 min in the past 4 weeks?” (Possible answers: 1 all the time/2 most of the time/3 sometimes/4 occasionally/5 never). The responses were collapsed into two categories, i.e., “absence of mental health symptoms” (“1” or “2”) and “presence of mental health symptoms” (“3”, “4”, or “5”).

Social relationships were measured by posing the following 3 questions: “During the past 4 weeks, how would you describe your relationship with your family/friends/neighbors?” (Possible answers: very good/ good /fair/ bad/ very bad). The responses were collapsed into two categories, i.e., favorable relationship (good, very good) and unfavorable relationship (fair, bad, very bad).

Activity-related limitations were measured by posing the following 4 questions: “During the past four weeks, did your health ever limit you in terms of these activities? If so, to what extent? Such activities included “riding a bike or doing homework for more than one hour”, “climbing three floors of stairs”, “bending, kneeling, or stooping”, “walking more than a mile”. The possible answers included the following: 1 not at all limited /2 limited a little /3 moderately limited /4 substantially limited /5 completely limited). The responses were collapsed into two categories: non-limitation (“1”) and limitation (“2”, “3”, “4”, or “5”).

Dependence on medical care was determined by posing the question “Are you currently taking medication or receiving medical treatment to help you carry out your daily life?” (Possible answers: not at all/ seldom/ sometimes/ often/ always). The responses were collapsed into three categories, i.e., not at all or seldom, sometimes, often or always. Sleep quality and appetite were assessed by posing the question, “How would you rate your sleep/appetite? Would you say, very good, good, fair, bad or very bad?” The responses were collapsed into three categories, i.e., good (very good, good), fair, and poor (bad, very bad).

Demographic and socioeconomic characters included age, sex, education (below primary school, primary school, secondary and higher), marital status (single, married, separated/ divorced/ widowed), living arrangements [living alone, living with other person(s)], and self-reported economic conditions and housing conditions (very good/ good /fair/ poor/ very poor). The responses were collapsed into three categories, i.e., good (very good, good), fair and poor (poor, very poor).

### Data analysis

The sample size was calculated according to the prevalence of the co-occurrence of physical, mental, and social problems among older people. On the basis of our exploratory research, the prevalence of the co-occurrence of physical health problems among older people was about 6.0%, and the sample size included 6,267 participants with a significance level of 5%.

The data included the mean and standard deviation continuous variables, and the frequency (%) of categorical variables. We constructed a series of hierarchical models using logistic regression analysis, with self-rated health as the dependent variable. The number of self-reported chronic diseases, mental health symptoms and poor social relationships, as well as their co-occurrence, were entered separately in step 1 (Model 1) and the demographic variables were entered in step 2 (Model 2). Housing conditions, living arrangement, appetite, sleep, medication, and activity-related limitations were entered sequentially in Model 3 and Model 4 in order to assess the unique contribution of each factor to self-rated health. The variables were selected by a step-wise method. *P* < 0.05 with a 95% CI of OR that excluded the null value of 1 was considered statistically significant. All analyses were conducted with SPSS 22.0 (SPSS Inc., Chicago, IL).

## Data Availability

The datasets generated and/or analyzed during the current study are available from the corresponding author on reasonable request.
